# A participatory action research approach to strengthening health managers’ capacity at district level in Eastern Uganda

**DOI:** 10.1186/s12961-017-0273-x

**Published:** 2017-12-28

**Authors:** Moses Tetui, Anna-Britt Coe, Anna-Karin Hurtig, Sara Bennett, Suzanne N. Kiwanuka, Asha George, Elizabeth Ekirapa Kiracho

**Affiliations:** 1 Makerere University School of Public Health (MakSPH), Makerere University, New Mulago Complex, P.O. B0X 7072, Kampala, Uganda; 2 Epidemiology and Global Health Unit, Department of Public Health and Clinical Medicine, Umeå University, 901 87 Umeå, Sweden; 3 Sociology Department, Umeå University, 901 87 Umeå, Sweden; 4 Department of International Health, Johns Hopkins Bloomberg School of Public Health, Johns Hopkins University, 615 North Wolfe Street, Baltimore, MD 21205 United States of America; 5 School of Public Health, University of the Western Cape, Robert Sobukwe Road, Bellville, 7535 Republic of South Africa

**Keywords:** District health managers, Health systems, Participatory action research, Competing values framework, Uganda

## Abstract

**Background:**

Many approaches to improving health managers’ capacity in poor countries, particularly those pursued by external agencies, employ non-participatory approaches and often seek to circumvent (rather than strengthen) weak public management structures. This limits opportunities for strengthening local health managers’ capacity, improving resource utilisation and enhancing service delivery. This study explored the contribution of a participatory action research approach to strengthening health managers’ capacity in Eastern Uganda.

**Methods:**

This was a qualitative study that used open-ended key informant interviews, combined with review of meeting minutes and observations to collect data. Both inductive and deductive thematic analysis was undertaken. The Competing Values Framework of organisational management functions guided the deductive process of analysis and the interpretation of the findings. The framework builds on four earlier models of management and regards them as complementary rather than conflicting, and identifies four managers’ capacities (collaborate, create, compete and control) by categorising them along two axes, one contrasting flexibility versus control and the other internal versus external organisational focus.

**Results:**

The findings indicate that the participatory action research approach enhanced health managers’ capacity to collaborate with others, be creative, attain goals and review progress. The enablers included expanded interaction spaces, encouragement of flexibility, empowerment of local managers, and the promotion of reflection and accountability. Tension and conflict across different management functions was apparent; for example, while there was a need to collaborate, maintaining control over processes was also needed. These tensions meant that managers needed to learn to simultaneously draw upon and use different capacities as reflected by the Competing Values Framework in order to maximise their effectiveness.

**Conclusions:**

Improved health manager capacity is essential if sustained improvements in health outcomes in low-income countries are to be attained. The expansion of interaction spaces, encouragement of flexibility, empowerment of local managers, and the promotion of reflection and accountability were the key means by which participatory action research strengthened health managers’ capacity. The participatory approach to implementation therefore created opportunities to strengthen health managers’ capacity.

**Electronic supplementary material:**

The online version of this article (doi:10.1186/s12961-017-0273-x) contains supplementary material, which is available to authorized users.

## Background

Participatory approaches are progressively being adopted to improve health managers’ capacity [1]. The concept of ‘learning health systems’ is now increasingly used to describe approaches that create partnerships among stakeholders and promote participation and learning [2]. However, in low-income countries, the reported contribution of participatory approaches to health interventions is largely restricted to community interventions and representation of communities on quality improvement committees [3–5]. Studies showing the use of participatory action research (PAR) to improve health managers’ capacity are limited. This study provided empirical knowledge on the contributions of PAR to enhancing health managers’ capacity in low-income countries.

According to WHO, health management is key and contributes direct benefits to the entire health system [6, 7]. Managers capacity-building is the process by which management capacities are learned or achieved to enable execution of key functions [8]. Central to managers functions are aspects of setting and achieving goals, problem solving, efficient use of resources and getting people to work together harmoniously [7, 9].

Managers capacity-building has mainly been approached through formal training programmes [10], which offer certain benefits but are not on their own sufficient to build the needed capacities for a health manager [10, 11]. On-the-job training, action learning and organisational experiences are some of the other complementary approaches for capacity-building [12, 13], each of which provide unique learning opportunities, but are also not devoid of challenges [12].

In low-income countries, management structures in the health sector are weak. The capacity of health managers at sub-national levels especially in rural districts, is even weaker [12]. Weak management undermines the performance of health systems despite widely available evidence on how to improve specific health outcomes [14]. The translation of this evidence into sustainable interventions at scale is often suboptimal. This is partly explained by limited local management capacity, other systems weaknesses and the heavy reliance on donor funding and external expertise [12, 15]. Such weaknesses usually attract local and international external agencies, including universities, non-governmental organisations and United Nations agencies, to remedy the situation.

The agencies habitually implement parallel projects to respond to specific health conditions such as malaria or HIV infections [16, 17]. They train staff and erect parallel structures to implement these time-limited projects [16]. While this registers short-lived achievements due to their reliance on external resources and expertise, continuity and scale up is seldom realised. Such external reliance undermines the development of local capacity, such as managers skills, which could be leveraged for continuity and possible scale-up [18]. Ironically, developing health managers’ capacity particularly at the sub-national level has often received relatively less attention, thus perpetuating health system weaknesses [19].

In Uganda, due to the decentralised form of governance, weaknesses in local health management capacity have worsened the already poor health outcomes in rural areas [20]. The local system is characterised by unresponsive local governments, low staffing levels, frequent stock-out of essential medicines and supplies, low motivation of workers, and a largely non-supportive work environment [21]. In addition, inadequately funded local governments are unable to respond to health managers’ capacity development needs, which further undermines the performance of the health system [17, 20]. Nonetheless, many research studies engage with districts in Uganda, thus presenting opportunities to learn how approaches to research could build local capacity and strengthen existing local health systems.

The Makerere University School of Public Health (MakSPH) is an example of a local external agency supporting the health system in Uganda through research studies. A research team at MakSPH used the PAR approach to improve maternal and neonatal health outcomes, which continue to remain poor in Uganda despite several interventions [22]. Further, the PAR approach offered opportunities to overcoming scale-up and sustainability challenges such as weak health management [23, 24]. PAR is a research approach that involves all relevant parties in collectively examining their current problematic situations in order to change and improve them [25, 26].

In this study, we explored the contributions of PAR to strengthening local health managers’ capacity. We used the Competing Values Framework (CVF) of management functions as an interpretative lens to examine the contribution of PAR to enhancing health managers’ capacity. The next two sections provide a brief description of the Maternal and Neonatal Implementation for Equitable Systems Project (MANIFEST) implemented by MakSPH using PAR and the CVF.

### The MANIFEST project and the PAR approach

The project was implemented in the districts of Kamuli, Pallisa and Kibuku found in eastern Uganda, from January 2012 to December 2015. These districts have a combined population size of more than 355,000 people [27]. The MANIFEST project aimed to contribute to a sustained reduction in maternal and neonatal mortality by using Gerald Susman’s PAR approach [25]. The approach has five main phases depicted in a cycle – problem identification, deduction of possible solutions, taking action, reflecting on the consequences of the actions and specifying learning. In 2012, during the formative stage of the project, different stakeholders were initially engaged to identify problems and solutions with regards to the poor maternal health outcomes in their respective districts.

After a series of participatory analysis and reflections, the intervention package agreed upon tackled both demand and supply side constraints to accessing maternal and newborn care [18]. In this paper, we focus on the supply side, in which supportive supervision, mentoring, training of health workers and managers, and recognition of good performing health workers were undertaken to improve the quality of health services. In addition, the PAR approach was used as a means of strengthening local capacity to improve on the quality of service delivery, which included improving health managers capacity. Indeed, health managers were part of the stakeholders that were initially engaged in identifying the problems affecting maternal health outcomes and they subsequently suggested and discussed the solutions, which informed the intervention package. In total, there were 42 health managers actively involved in implementation of the MANIFEST project in different capacities across the three districts during the different phases of PAR. For example, at the phase of taking action, select district level health managers were at the forefront of the coordination and implementation of the project activities, supported by health facility health managers at sub-county levels.

To undertake the reflection and learning, quarterly review meetings (from June 2013 to April 2015) were held at district, sub-county and community levels. Table 1 provides the details of stakeholders involved in the project review activities at different levels. Different persons led the reflection and learning processes during these meetings at different levels. MANIFEST project team members actively supported these meetings to promote learning. The PAR cycle was repeated on a quarterly basis with a refinement of issues to be handled or new ones. More details of the PAR approach as used in the MANIFEST study have been published elsewhere [24, 28]. Herein, we explored the contributions of PAR to strengthening the health managers’ capacity.

**Table 1:** Participants, facilitators and support teams of the project activities at different levels

Level of implementation/review meetings	Stakeholders/participants	Lead facilitators	Support teams
District level: District implementation committee meetings	Health managers, political heads, assistant district health managers, district senior nursing officers, health educators, biostatisticians, community development officers, secretaries for health to the district councils, resident district commissioners, chief administrative officers, heads of health sub-districts or hospital managers, internal security officers, health information officers, a representative from the NGO forum, local religious leaders, and health accountants	Health managers, senior nursing officers, political and administrative heads of the districts	At least four members of the MakSPH project team; the project team attended all the meetings every quarter
Sub-county level: Sub-county implementation committee meetings	Administrative and political heads of the sub-counties, secretaries for health to the sub-county council, health centre III managers, community development officers, health assistants, and local religious leaders	Administrative and political heads of the sub-counties	Two district level supervisors (managers), who were members of the DHT Two members of the MakSPH project team; the project team attended all the meetings every quarter
Community level: VHT group meetings	VHTs in a particular sub-county; there were 850 villages in the intervention area; every village had 2 VHTs; each sub-county held 1 or 2 meetings depending on the number of VHTs	Health workers, facility health managers and health assistants who provided supervisory oversight to the VHTs	Two district level supervisors (managers), who were members of the DHT Two members of the MakSPH project team; the project team attended at least 12 VHT group meetings every quarter

*DHT* district health team, *MakSPH* Makerere University School of Public Health, *VHT* village health team

### The MANIFEST implementation context

**Fig. 1:**
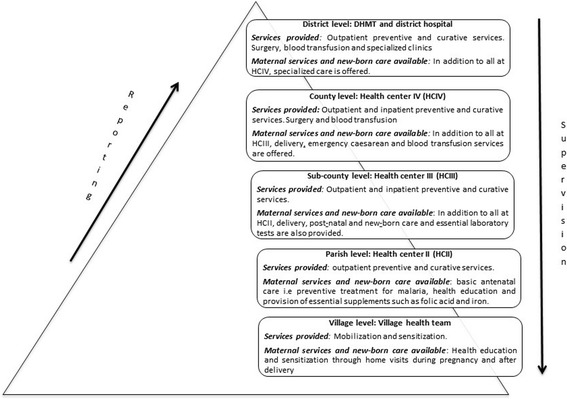
Organisation of health services at district level in Uganda

The intervention package was implemented using the decentralised district structures and resources (Fig. 1) [29]. The use of existing structures was meant to strengthen them so as to increase chances of continuity and sustained capacity improvements. At district level, the health department ensures the delivery of health services. Under the MANIFEST project, the health department coordinated and implemented district level project activities. The department is typically comprised of about 15 members technically referred to as the district health team (DHT). The DHT is headed by a district health officer (DHO) and is supported by a number of other health staff, including the district health educator, the district heath inspector, focal persons for different programmes such as immunisation and maternal/child health, and the district biostatistician. The health department was supported by the District Health Management Team, which is also headed by the DHO. The District Health Management Team, in addition to DHT members, includes health facility managers, politicians, administrators, and representatives from local non-governmental organisations, the private sector, other departments and external agencies.

The DHO is usually a medical officer with postgraduate training in public health. While the DHO is formally employed to undertake managerial duties, they might also practice clinical medicine, especially in rural districts where attracting medical officers is a challenge. The other DHT members hold different qualifications, including nursing, environmental health sciences and biostatistics. They often have no management training at the time of appointment to these offices, but, owing to their senior positions, they sometimes benefit from limited training opportunities supported by the local governments to improve their management skills [30]. Below the DHT are service delivery facilities, which are usually organised into a health sub-district comprising a number of health facilities within a specific catchment area.

Figure 1 provides a summary of the services offered at each level of care. Each of the service points from the parish level upwards are managed by a health facility manager, who works with a team of other health workers organised into departments. The organisational and management aspects get more complex at each higher level of care. Facility managers (except for hospital managers) undertake managerial roles in addition to their formal clinical or nursing roles, as additional assignments rather than official appointments [30]. This further exacerbates management weaknesses as it creates a low sense of commitment to the role and attracts less attention for capacity-building. The facility managers offered oversight to the project activities and were involved in implementation of project activities at sub county level.

Health managers at various levels are often constrained by budgetary limitations and inadequate skill sets to navigate systems challenges. Managers, just like other civil servants at the district level, often operate within a *laissez faire* environment and attitude characterised by low commitment to quality, limited availability at work stations and low motivation [21, 31]. In addition, there is a generally poor linkage among different local government structures and levels, and a high level of dependence on external agencies to implement projects.

Finally, external agencies often provide support to the districts ranging from one-off trainings to the actual provision of specific services. MakSPH, as an external agency, implemented the MANIFEST intervention within the decentralised structures of the districts by providing technical guidance and funding project-activities in a participatory manner. MakSPH was involved in training of local stakeholders to implement project activities, participated in activity implementation, review meetings, monitoring and quality assurance.

### Conceptual framework – CVF

**Fig. 2:**
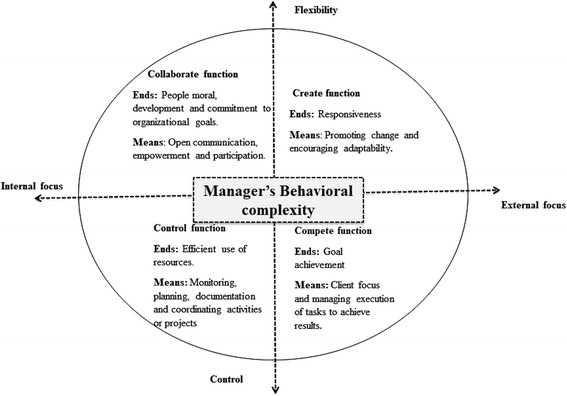
The competing values framework

The CVF (Fig. 2) was chosen first because it is a complex representation of earlier models of management functions, i.e. the rational goal, internal processes, human relations and the open systems models [32]. Each of these emphasised a limited set of functions; however, CVF asserts that all four models, although differing in focus, should be viewed as necessary and complementary in modern times [32]. Secondly, the framework has mainly been used to undertake quantitative assessments of organisational cultures [33, 34]. Qualitative inquiry enhances the use of the CVF by revealing the complexities in management functions.

CVF identifies four manager capacities (collaborating, creating, competing and controlling) by categorising them across two axes, one contrasting flexibility versus control, and the other internal versus external focus. The categorisation is a demonstration of the complex, not mutually exclusive, managers’ capacities. The framework further asserts that managers need to develop behaviour complexity, which is the ability to simultaneously draw upon and use the four different capacities reflected in the framework at any one time as a measure of effectiveness [9].

Under the ‘collaborate’ function of management, the aim is to maximise workers’ morale, development and commitment to organisational goals. Skills for open communication, people empowerment and participation are needed to achieve this [32]. The ‘create’ function aims at promoting responsiveness to clients’ needs. To achieve this, a focus on the ability to promote change, be adaptable and respond to client needs is required. In the ‘compete’ function, the objective is to achieve set goals. Managers are encouraged to support their teams in building a shared vision for client satisfaction and to manage the execution of tasks to achieve set goals [32]. Finally, the ‘control’ function seeks to ensure an efficient use of resources, emphasising the need for planning, monitoring, documentation and coordination of activities [35].

## Methods

### Study design, selection of informants and data collection techniques

We undertook a qualitative study that explored the management skills gained by health managers engaged through a PAR approach to implement the MANIFEST project. Herein, we focused on district level and health facility managers.

A total of 16 purposively selected managers were interviewed individually, including seven district level health managers and nine health facility managers across the three intervention districts. These were purposively selected because of their direct management responsibilities and involvement in the implementation of MANIFEST project. Our sampling approach ensured the maximum variation principle of qualitative research [36]. The district level health managers were the lead coordinators of the MANIFEST activities and were responsible for planning, scheduling and leading the review of activities at the district levels and supervising those at sub-county level. At the sub-county level, the health facility managers played three main roles, namely overseeing the implementation of community level activities such as community dialogues, home visits by village health teams (VHTs) and supervising the VHTs. In addition, they represented the health facility at the sub-county level quarterly review meetings.

To collect the data, an open-ended interview guide was used to allow for flexibility and to create a good level of rapport with the informants. All interviews started with sensitising concepts [37] from the PAR and management literature such as collaborating with others, reflecting on actions, fostering new ideas, planning and leading others. While these guided the discussion, care was taken to remain open to emerging directions of the interviews and probing was undertaken following the responses of the informants. On average, the interviews lasted 45 minutes each. All interviews were digitally recorded and later transcribed by a research assistant whose work was checked for consistency by the first author (MT). These interviews were conducted in August 2015, which was the last year of the MANIFEST intervention.

In addition, minutes taken from the two quarterly review meetings of the different stakeholders at district and sub-county levels were reviewed to supplement the interview material. These were deemed to provide a wider scope of issues tackled during the entire implementation period of the project. Review meetings were planned and happened in eight out of the 12 quarters in which the MANIFEST intervention was implemented. In addition, reflections from the earlier meetings as well as other project activities and interactions were captured through the participant observation undertaken by MT.

The quarterly meetings at district level had a minimum of 15 participants who formed the district implementation committee for the intervention. At sub-county level, a similar committee was comprised of at least seven persons in each of the 27 sub-counties in which MANIFEST was implemented. The sub-county quarterly review meetings combined two or three sub-counties at each venue mainly to foster learning across sub-counties but also for logistics. The participants in these meetings are detailed in Table 1. The different data sources served the purpose of triangulation of study findings; a technique that is synonymous with qualitative research [38]. Table 2 is an illustration of the data sources.

**Table 2:** Data sources for the study

Kind of data source	District level	Health facility level	Total
Interviews with Health managers	7	9	
Total			16
	District level	Sub-county level	
Quarterly review meeting minutes from the 1st and 2nd quarters of 2015	2 from each district	2 from 2 sub-county level review meetings in each district	
Totals	6	12	18
Participant observation notes from review meetings and other project activities at regular quarterly intervals for a period of 3 years (2013–2015)	The first author was actively involved in monitoring and offering support during the implementation process to the district stakeholders and usually made reflective memos, which informed the analysis process		

### Data analysis approach

An inductive and deductive approach to thematic analysis was adopted [39]. The process started during the data collection, where reflection and memo taking were applied to identify points of saturation [39]. At interview 15, the themes started to repeat; however, one more interview was added to ensure complete saturation. After the data was transcribed, it was exported into MAXQDA for Mac version 11.2.1. Next, the transcripts were read and re-read by MT to get familar with the dataset.

An inductive open coding process followed; at this stage, the reflective process continued, where MT was careful to remain open to the data and themes that arose. To aid this process, the codes were shared with co-authors (ABC, AKH, SK and EEK) for review and interpretation in an iterative process that involved going back to the transcripts and recodings.

Thereafter, grouping the codes that shared common aspects was undertaken to form categories guided by the four management capacities of the CVF through a deductive analysis process [32]. The categories were further developed into four themes by identifying linkages and relationships between them. Similarly, looking for both confirmation and deviance from themes continued through the deductive thematic analysis process. This was undertaken by using a data extraction table that aided in the review of the meeting minutes and observations. In general, the meeting minutes and memos confirmed and enriched the interview findings. For example, action points relating to some of the creative innovations expressed by the informants were searched for and linked to the management skills that were reported to have improved. Finally, crosschecking of the themes against open codes and transcripts was undertaken to ensure connectedness of themes to the transcripts [39].

## Results

**Fig. 3:**
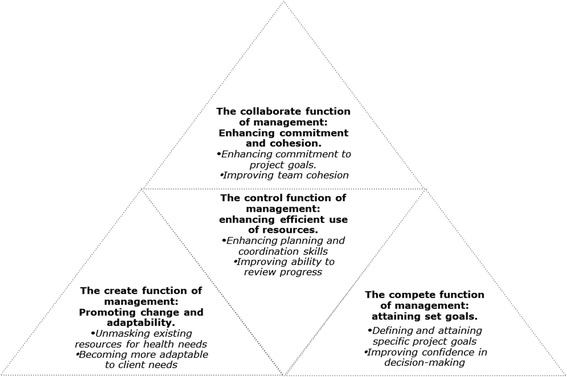
Boundless management functions enhanced by PAR

The themes developed are presented according to the functions of management articulated by the CVF [32, 40]. To supplement the findings from the interviews, reflections from the observations and meeting minutes related to the four management capacities were added. Figure 3 demonstrates the boundless nature of the four management functions found to have been enhanced in this study. The PAR approach enabled the health managers to simultaneously develop their skills to collaborate, create, compete and control depicted by the dotted lines along the pyramid, making a manager more effective in today’s dynamic and complex world.

### The ‘collaborate’ function of management: enhancing commitment and cohesion

Generally, the managers noted that the PAR approach, although challenging in some instances, enabled greater commitment and teamwork.

#### Enhancing commitment to project goals

The managers observed that commitment to project goals strengthened over time mainly due to free and open dialogue across the different stakeholders detailed in Table 1. Open dialogues were inherent in the design of the MANIFEST project. These highly interactive open discussions were useful in challenging the health managers to constantly improve on their communication skills. This yielded trust among stakeholders (Table 3), which according to the CVF, is essential for building morale and empowering workers, and hence a source of increasing commitment to project goals.

An increased sense of local ownership of the project, contributions toward project goals and reciprocal relationships among stakeholders further illustrated the participation and empowerment of the different players. In Table 3, an illustration of increased commitment to continue project activities was observed by the end of the project. A health facility manager shared his reflection of greater stakeholder commitment achieved:

“*People have really owned this project. You see the politicians these days encouraging pregnant women to save for transport costs at burials. Then last time at the meeting, the Chairman* [political head of a sub-county] *was really challenging us to plan for the tricycle and members were responding positively….*” (Health facility manager 1)

#### Improving team cohesion

The health managers noted that MANIFEST provided opportunities for politicians, health workers, religious leaders, health managers and administrators to work together. This was illustrated by an improvement of teamwork and involvement of several stakeholders in implementing project activities. This enabled managers to learn the dynamics of team building by practicing it. A facility manager recounted the benefits of actively participating in the implementation processes of the project in the quote below:

“*We are now doing monthly staff meetings and then allocating responsibility to different people. This has been very helpful in bringing us together. You see with MANIFEST, you have to meet, there is no way you can do all that work alone. I remember I used to just come here once in a while and tell people what to do and it was difficult to get them to work. Now, I understand people better, the challenges here and people are usually happy to share in the meetings.*” (Health facility manager 7).

Enhancing commitment to project goals and improving team cohesion was nonetheless challenging. The norm of working in isolation of others was difficult to overcome. This was because different stakeholders had not been actively working together to implement activities prior to MANIFEST. Working in isolation was viewed as a means of being in control of one’s sphere of influence, which then created conflict with the need to collaborate as a management function. The desire for specific health managers to implement and coordinate all the activities by themselves was observed to have been high initially, but the need for teamwork grew with time (Table 3).

This nonetheless presented an opportunity for the managers to strike a balance between the ‘collaborate’ and ‘control’ functions of management, a concept the CVF refers to as behavioural complexity. The managers interviewed reckoned that the tensions between the two management functions, while disruptive at times, actually enabled them to better appreciate the differing needs and contributions from others. For example, the managers made deliberate efforts to involve different stakeholders, such as politicians, in project activities as they came to a better appreciation of their roles and needs.

### The ‘create’ function of management: promoting change and adaptability

The ‘create’ function of management presents the managers’ abilities to unmask existing resources for health needs and their adaptability skills. We observed that creativity among the health managers improved over time as they implemented different activities. However, the desire to maintain the status quo was also observed to have countered creativity in some cases (Table 3).

#### Unmasking existing resources for health needs

The managers’ ability to identify existing resources for health was enhanced through opportunities for collectively identifying problems and solutions, reflecting on actions and learning. The MANIFEST project challenged managers at different levels to continuously explore their own rather than external resources. To achieve this, the MakSPH project team consciously maintained a supportive role during the implementation of the project activities in order to foster identification of local ideas and resources. For example, one district health manager boasted of how he was challenged to set up a data centre for the district to support evidence-based decision-making processes. In other cases, facility level managers spoke about making better use of storage spaces, enhancing their negotiation and partnerships creation skills:

“*My store was a mess before, it had drugs, books, other supplies, etc., it was in such a mess for a long time and we did nothing about it. I had thought that we just need a bigger store. So, one time the MANIFEST supervisors came here and I was embarrassed, they challenged me, we went to the store and started organizing it together, they opened my eyes, we now have a lot of space in the store.*” (Health facility manager 9)

“*I have shared with the district about the lighting problem but we have not got any solution yet. But during the last quarterly review meeting, I was challenged about doing something as a manager and now we have put aside some money every month from our quarterly allocations to clear the electricity bill. We talked to Umeme (power distribution company) and they accepted to reconnect us and we shall clear the bill with time.*” (Health facility manager 2)

#### Becoming more adaptable to client needs

Opportunities of working with others increased the managers’ awareness of their clients’ needs, hence challenging them to be more adaptable to situations and needs. In addition, feedback linkages facilitated managers’ responsiveness. For example, better facility-opening hours, attitudes of health workers and shorter waiting-times for patients were noted following feedback from communities. Similarly, managers used improved awareness of community resources to continuously challenge communities to save for their health needs by pointing them to existing savings groups. One district health manager, recounted the following regarding improved adaptability and responsiveness to client needs:

“*We started receiving reports that the mothers leave the health centre very late on antenatal clinic days. Then when we had a stakeholder meeting, the same issue came up and the politicians were saying the communities are not happy. So, we reviewed this in one of our meetings and now we have antenatal clinics every day of the week, so that the work load is spread.*” (District health manager 3)

However, sometimes the desire to maintain the status quo among the health managers conflicted with creativity aspirations, which exemplified a conflicting situation between the ‘control’ and ‘create’ functions of management. While the MANIFEST project encouraged creativity through its design (flexibility and local championing), the dependency on the MakSPH research team by the health managers was a continuous struggle (Table 3). In addition, breaking out of a ‘comfort zone’ to become more responsive by collaborating with others was an unremitting negotiation.

Review meetings at district and sub-county levels were useful for striking a balance between being innovative and maintaining the status quo. The different stakeholders were able to better appreciate their needs, interests and contributions within their means. MakSPH maintained its facilitating role, which allowed and continued to challenge the local stakeholders to further explore their own local solutions and contributions. This yielded some benefits as one facility manager remarked:

“*Who ever knew that the sub-county could mobilize resources to procure a motorcycle ambulance? Sincerely MANIFEST has opened our eyes.*” (Health facility manager 4)

**Table 3:** Action points, observations and their status related to the ‘collaborate’ and ‘create’ functions of management from quarterly review meeting minutes

Management functions	Action points, observations and their status from quarterly review meetings and other project activities across the project implementation period		
	2013 (2 quarters)	2014 (2 quarters)	2015 (2 quarters)
Collaborate: Promoting open communication empowerment of others and stakeholder participation skills	Orientation of different stakeholders, and forming of work teams was observed and acted upon, e.g. community development officers were empowered and actively engaged. However, fear and anxiety during meetings was observed in the first two quarterly review meetings since stakeholders with different power relations were involved; a tendency to ‘let things be’ was noted	Free and open discussions between stakeholders started to improve with time, increasing stakeholder buy in, trust and commitment were noted. Growth of teamwork in implementation of project activities was observed across stakeholders; nonetheless, some stakeholders (e.g. politicians) were observed to have remained suspicious of health managers in particular	Sharing of ideas and identification of local resources was noted. An increased sense of political responsibility and trust between stakeholders was stronger Discussions about how to continue implementing the project activities started in the last quarter of 2014 and continued in the whole of 2015
Create: Promoting change and encouraging adaptability	Limited generation of local ideas and solutions was observed; a high dependency on the MakSPH project team members was notable Stakeholders were careful not to ‘step on each other’s feet’ when challenging the status quo They were not sure of how much to trust the Project team members in attendance of the review meetings. Rigid mind sets about usual procedures, constraints and limitations observed. A low willingness to change was also observed initially	With increased stakeholder trust and commitment, free brain storming of local ideas and testing them out started and was sustained throughout the year The desire to cause change through critical thinking begun to grow, especially at district level, lead by the district health officers; for example, health worker motivation was debated and embedded in the district plans Discussions of maternal health issues such as death audits and health worker discipline was started	Prioritising maternal health in budgeting was improved; for example, sub-counties begun to budget for VHT incentives as well as motorcycle ambulances; at least one sub-county actually bought a motorcycle ambulance for referral purposes by the end of the project, while several others engaged politicians who donated motorised ambulances

### The ‘compete’ function of management: attaining set goals

In the ‘compete’ function, the health managers’ skills in defining and attaining goals and their improved confidence in decision-making are presented. Health managers perceived their goal definition and decision-making skills to have been enhanced during the MANIFEST project.

#### Defining and attaining specific project goals

Improving service availability, attitudes of health workers, quality of care, accountability and saving the lives of mothers and newborns were some of the targets that the health managers had under the MANIFEST intervention. The managers, together with the other stakeholders, set these targets as a response to clients’ needs (external focus). Consequently, the health managers boasted of having attained specific goals as a result of their goal clarification (control), which necessitated deliberate actions taken by different stakeholders to achieve them. Such achievements included construction of a number of infrastructural projects such as placenta pits, pit latrines and waiting sheds (Table 4). A district health manager remarked:

“*Health centre A had no pit latrine for patients, the issue was debated upon during the sub-county review meeting, action was taken and money was allocated at the sub-county for constructing the pit latrine.*” (District Health manager 7)

In recognition of achievements made by the health workers in providing better services to the population, a facility manager commented:

“*To me now I see that there is less absenteeism in the facilities than before. Then, I think there is also a great improvement in the attitude of the health workers, mothers are no longer complaining of being abused by health workers.*” (Health facility manager 1)

To achieve the set targets, managers had to work with several stakeholders, which required flexibility. Flexibility is synonymous with the ‘collaborate’ and ‘create’ functions rather than the ‘compete’ and ‘control’ functions of management. This provided an opportunity for managers to learn how to balance management functions and make trade-offs where necessary, thus demonstrating behavioural complexity. Managers were hence able to define their goals to meet clients’ needs (external focus) and follow-up on specific actions (control) to ensure achievements. They did this while at the same time creating spaces for stakeholder collaboration (internal focus) and creativity by stimulating the respectful sharing of multiple opinions and ideas (flexibility).

#### Improving confidence in decision-making

Managers reported that the process of strategic and analytical thinking enhanced their confidence in decision taking. Through the reflective process of PAR, health managers had the opportunity to reflect upon their priorities, problems, options and resources. This improved the quality of their decisions, supported by commonly set goals. These skills, according to the CVF, are useful in developing capacity that enables the attainment of desired goals. One district level health manager, while referring to his enhanced confidence, made the following remarks:

“*I am now more confident of myself, I take decisions because I know I will get the support of others since we have the same goal. So, when we want to reallocate our resources to a pressing need, I simply communicate my concerns to others, those days I used to fear what they will say. So, at times we could send back money to the centre.*” (District health manager 1)

### The ‘control’ function of management: enhancing efficient use of resources

The health managers’ ability to efficiently use available resources was viewed in two ways, namely as planning and coordination of activities and reviewing of progress. Planning was viewed as a process by which managers reflected upon their resources and how to maximise their benefit. Coordination facilitated organisational skills for resources to work in sync. These skills were noted to have improved, although to a lesser extent compared to the skills in the previous functions presented (Table 4).

#### Enhancing planning and coordination skills

The MANIFEST project involved managers in the planning, budgeting, coordination and implementation of activities. This was an opportunity for the health managers to challenge their skill-set by putting into practice these standard management processes.

Specifically, the health managers scheduled activities, put together implementation teams, budgeted and accounted for resources used for the implementation of project activities at the district level. The managers thought their planning capacity had generally been boosted by the opportunity to coordinate MANIFEST activities as noted in the quote below by a district level health manager:

“*As we talk now, as a district I think we have gotten more capacity in planning and also coordination of activities by being directly involved and leading these activities. We control the budget and we are also leading the process of scheduling the different activities at our level.*” (District health manager 6)

However, some managers felt that they had not fully exploited the opportunity to develop their planning and coordination skills. MT indeed observed a limited sense of preparedness for project activities among some managers (Table 4). Delays in scheduling activities, accounting for resources and mobilisation of participants for activities, were noted. This was usually related to the heavy dependence on MakSPH for coordination guidance, the managers’ limited experience in planning and coordination of projects, and the competing demands that managers had. The quote below, taken from a district level health manager, exemplifies the differing opinion on the enhancement of planning and coordination skills.

“*The planning skills, I would say yes and no, yes we have been mobilizing and scheduling the activities but this project is big, so I would say no, we have not improved much in planning. I don’t think we have had very strong support from the leaders like the DHO. They are very busy with other administrative issues, so a gap was left. We don’t really have regularly planning meetings, I am sorry to say this but it would be good to sit as a team and say we are now going to develop a plan for this activity, which is not happening much.*” (District health manager 2)

#### Improving ability to review progress

The MANIFEST project involved managers in monitoring activities, report writing and quarterly review meetings. These processes helped managers develop a culture of progress review, improved report-writing skills, strengthened the documentation of health events such as maternal deaths and enriched the usage of health data for decision-making (Table 4). Improvements in budgeting and accountability were also widely noted by the health managers. A facility health manager reflected,

“*Actually, before MANIFEST, I would hold meetings without even ever reading the previous minutes. But in those sub-county meetings, I have learnt that I need to be accountable, because everyone is like, ‘but we agreed on that last time, what have you done?’ Yeah, it is helping me because reviewing what you discussed helps you understand how much you have achieved and sometimes you identify the obstacles hindering you.*” (Health facility manager 3).

Involvement in project activities also enhanced a sense of responsibility among health managers, which created a need to actively be acquainted with processes and procedures of the project.

Nonetheless, it was observed that managers were initially less concerned about reviewing progress of project activities since external agencies would normally be in control (Table 4). The lead coordinators of the project often felt overworked and sentiments of MANIFEST being stressful were noted.

While the need to constantly be acquainted with the processes and procedures of the project could at times be perceived as stressful given other routine roles, some managers viewed such processes as beneficial for better service delivery. One district level manager, in reference to the ‘new’ experience of being in-charge of project coordination, mentioned:

“*I am so tired, this project is really busy, I have never been so engaged. Anyway, much as it’s difficult, I think I am really learning a lot of things which we usually take for granted, like accounting for money spent, I thought it was easy but you have to plan in advance and ensure that people spend the money as budgeted.*” (District health manager 2)

**Table 4:** Action points, observations and their status related to the ‘compete’ and ‘control’ functions of management from quarterly review meeting minutes

Management functions	Action points, observations and their status from quarterly review meetings and other project activities across the project implementation period		
	2013 (2 quarters)	2014 (2 quarters)	2015 (2 quarters)
Compete function: client focus and managing execution of tasks to achieve results	The focus on improving the quality of maternal health services offered was initially observed to have been generally low; stakeholders, including managers, accepted the status quo and viewed the MANIFEST project as any other that will end without lasting effects	Improving confidence among stakeholders in the ability to cause change was observed; following up on commitments from stakeholders and being more responsible increased in 2014; however, a sense of dependency and need for more support in execution of tasks was still prevalent even through 2015; some stakeholders continued to lack a sense of clarity on their roles even in 2015	Building of staff houses, placenta pits, starting data centres and fencing off facility land, were noted as some of the achievements Discipline and monitoring of health worker availability was observed to have improved
Control function: monitoring, planning, documentation and coordinating activities or projects	Reviewing of progress was observed to be missing; during the second review meetings in 2013, minutes from the previous meetings were generally not available and members had limited recall of any action points agreed upon; planning and coordination of activities was observed to have been heavily reliant on the MakSPH project team	A commitment to assign specific persons to take minutes was reaffirmed and followed through the year; members then started to review progress against their action points in every subsequent meeting. Teams to plan as well as focal persons for each of the project activity sets or components were formed; nonetheless it was observed that as prior planning of activities was often emphasised in meetings, this was implemented sporadically; MakSPH project team still played a central coordination role	Integration of project review meetings into mainstream meetings was started; review of maternal health issues was uplifted to council meetings at both sub-county and district levels A quarterly District Health Management Team was the other avenue through which project activities were reviewed Planning and coordination of activities was observed to have improved although still dependent on MakSPH project team to a large extent

## Discussion

This study presented our findings according to the four CVF management functions [32], namely the ‘collaborate’ function of management – enhancing commitment and cohesion; the ‘create’ function of management – promoting change and adaptability; the ‘compete’ function of management – attaining set goals; and the ‘control’ function of management – enhancing efficient use of resources.

This study found that the PAR approach enhanced the management functions in four main ways, namely by expanding the managers interaction space, encouraging a flexible approach to research and implementation, empowering managers to plan, coordinate and implement project activities, and promoting their analytical reflection and accountability skills.

First of all, the PAR approach, through its wide stakeholder involvement, expanded interaction space among players in the health system at district level. According to the CVF, involvement of others through open communication, mentoring and team building is essential for morale, commitment and people development, which are the focus of the ‘collaborate’ function of management. Participation of different stakeholders (Table 1) in an intervention led to motivation, the creation of a sense of local ownership and generated commitment to common courses of action [41]. On the other hand, non-participatory approaches often create a sense of dependency on expert knowledge and solutions, hence limiting workers morale, personal development and commitment to organisational goals [3, 42]. However, the use of participatory approaches has to be matched by organisational level commitment to facilitate collaboration, otherwise it becomes demotivating when managers fail to positively interact with stakeholders with varying power positions such as local government officials [3].

Secondly, flexibility boosted the ‘create’ function of management. Managers reported that participation and flexibility promoted interaction, which nurtured local ideas and novelty. In addition, the wide stakeholder involvement synonymous with flexibility enabled managers to identify client needs and to be responsive, therefore cultivating a favourable work environment for themselves [43]. This is a rare attribute of non-participatory approaches that usually have a heavy reliance on external knowledge and expertise [18]. Moreover, limited engagement of stakeholders in externally controlled projects makes adjustments to protocols and procedures to meet dynamic client needs difficult. Notwithstanding, some managers indeed prefer the ‘clarity’ that comes with non-participatory projects [44]. While strict protocols are useful for testing hypotheses, approaches that allow learning in the ‘real’ and dynamic world could be more appropriate for proven interventions and systems research [5].

Thirdly, the PAR approach improved managers’ skills to plan, coordinate and implement project activities. It is imperative to note that management capacity transcends simple possession of knowledge (which can be acquired through training) to include behavioural capacity for appropriate application [32]. From the managers’ perspectives, the PAR approach provided opportunities to practice theoretical knowledge on planning, coordination and implementation of activities. This was found to be essential in enabling managers to enhance key management capacities within the ‘compete’ and ‘control’ functions of management, which are a vital set of skills for circumventing the different layers of authority and power to achieve goals and ensure efficiency, respectively [45, 46].

Lastly, the PAR approach enhanced analytical reflection and accountability skills among health mangers. The approach promoted the local scheduling of activities, budgeting, execution and accountability of and for project activities and funds, respectively. In addition, the review phase of the PAR cycle strengthened monitoring and advanced the development of a progress review culture. These features of the PAR approach where useful in enabling health managers to develop skills relevant for the 'control' capacities of management such as documentation, planning of activities and monitoring.

### Methodological considerations

The study results have to be interpreted in the context of key methodological considerations. Firstly, MT conducted the interviews and observations for this study; he has a masters’ degree in Public Health and served as a coordinator for  the MANIFEST project. He had at least 8 years of implementation research experience by the study time, which involved active engagement of district level health systems to improve their responsiveness to the populations. Therefore, he had an established relationship with the informants of this study prior to the interviews. Two other authors (EEK and SNK) of this paper were also actively involved in the implementation of MANIFEST. These dual roles indeed influenced the manner in which the study was conducted. Being ‘insiders’, this enabled MT, EEK and SNK to adequately understand and describe the context within which the study results were obtained and interpreted. Further, with the established trust with the informants, MT, who had worked for over 8 years in different research roles, was able to openly discuss both the desirable and undesirable outcomes of the project.

Secondly, at the start of the MANIFEST project, the purpose of the intervention was jointly reached between the researchers and the local stakeholders. Similarly, while undertaking this specific study, since it was embedded in the MANIFEST project, specific communication regarding the purpose of the study was given by MT. The informants were made aware of the goal to contribute knowledge relevant for the strengthening of health managers’ capacity at district level. With this goal, MT held the assumption that PAR provided opportunities to strengthen health managers’ capacity, which definitely which could have biased him to think of PAR only in positive ways. Being aware of this possibility prepared MT to respond accordingly. For instances where a tendency to report only desirable outcomes was noted, working with authors (ABC, AKH, SB, AG) not directly involved in MANIFEST minimised such influences, thereby increasing the credibility of the study. Additionally, as noted in the methods section, we inductively studied PAR’s ability to strengthen management capacity as our initial conception and then abduction was applied to compare it to the CVF on management functions. Lastly, the use of different methods enabled us to triangulate our findings and thereby strengthened the deductions made [47].

Thirdly, interviews from other stakeholders working with the health managers such as politicians and administrators, would have enriched the health managers’ perceptions of their enhanced capacity. The stakeholder-wide review of meeting-minutes and observations made by MT nonetheless served to supplement and triangulate the findings from the managers’ interviews.

## Conclusions and recommendations

Strong health management systems are core to the strengthening of health systems in low-income countries; however, health managers’ capacity, as well as the approaches used to strengthen it, are often weak especially at sub-national levels. Good management will ultimately ensure better resource utilisation. The use of a PAR approach strengthened local managers’ capacity, which enhanced chances for continuity of the health improvements. Consequently, this study harnessed the contributions that participatory approaches to health interventions could offer to enhance district health managers’ capacity.

Managers’ abilities to collaborate with others and be creative were enabled through expanded interaction spaces, openness, practice opportunities, promotion of local ideas and solutions, and challenging of the status quo. On the other hand, the ‘compete’ and ‘control’ functions of management were enhanced mainly through the empowerment of managers to better plan, coordinate and implement activities. In addition, opportunities to monitor and review project activities sharpened the managers’ capacity in analytical reflection and accountability skills. Building managers’ capacity is thus a complex phenomenon that requires different perspectives and stakeholders working in synergy, notwithstanding emerging conflict.

It is therefore recommended that external researchers intervening in the health sector use participatory approaches especially when undertaking implementation and health systems research that seeks to answer complex questions. Such approaches create opportunities to strengthen local structures such as management, which are essential for a sustained improvement in health outcomes. The use of participatory approaches should nonetheless be adopted with caution as other studies found them time-intensive, and requiring prolonged engagement and organisational level commitment. They also often challenge institutional cultures and can initially create strain and conflict; however, as noted above, this may be essential for creating needed changes. The use of non-participatory approaches is nonetheless relevant, especially for experimental studies that require strict control of procedures to prove concepts and ideas.

### Open peer review

Peer review reports for this article are available in Additional file 1.

## Additional file

Additional file 1: Open peer review reports. (DOCX 21 kb)

## References

[1] Mshelia C, Huss R, Mirzoev T, Elsey H, Baine SO, Aikins M (2013). Can action research strengthen district health management and improve health workforce performance? A research protocol. BMJ Open.

[2] Greene SM, Reid RJ, Larson EB (2012). Implementing the learning health system: from concept to action. Ann Intern Med.

[3] Bradley JE, Mayfield MV, Mehta MP, Rukonge A (2002). Participatory evaluation of reproductive health care quality in developing countries. Soc Sci Med.

[4] Haaland A, Vlassoff C (2001). Introducing Health Workers for Change: from transformation theory to health systems in developing countries. Heal Policy Plan.

[5] Keusch GT, Kilama WL, Moon S, Szlezák NA, Michaud CM (2010). The Global Health System: linking knowledge with action—learning from malaria. PLoS Med.

[6] Prashanth N, Marchal B, Devadasan N, Kegels G, Criel B (2014). Advancing the application of systems thinking in health: a realist evaluation of a capacity building programme for district managers in Tumkur. India Health Res Policy Syst.

[7] World Health Organization. Towards better Leadership and Management in Health. 2007. http://www.who.int/management/working_paper_10_en_opt.pdf Accessed 14 Apr 2014.

[8] Prashanth NS, Marchal B, Criel B (2013). Evaluating healthcare interventions: answering the “How” question. Indian Anthr.

[9] Hooijberg R, Quinn ER, Phillips RL, Hunt JG (1992). Behavioral complexity and the development of effective managers. Strategic Leadership: A Multiorganizational-Level Perspective.

[10] Egger D, Ollier E, World Health Organization, Department for Health Policy, Development and Services (2007). Managing the Health Millennium Development Goals: The Challenge of Management Strengthening: Lessons from Three Countries.

[11] Rowe L, Brillant S, Cleveland E, Dahn B, Ramanadhan S, Podesta M (2010). Building capacity in health facility management: guiding principles for skills transfer in Liberia. Hum Resour Health.

[12] Dorros GL (2006). Building Management Capacity to Rapidly Scale Up Health Services and Health Outcomes.

[13] Crisp BR, Swerissen H, Duckett SJ (2000). Four approaches to capacity building in health: consequences for measurement and accountability. Heal Promot Int.

[14] Travis P, Bennett S, Haines A (2004). Overcoming health-systems constraints to achieve the millennium development goals. Lancet.

[15] Katz I, Glandon D, Wong W, Kargbo B, Ombam R, Singh S (2014). Lessons learned from stakeholder-driven sustainability analysis of six national HIV programmes. Health Policy Plan.

[16] Adam T, de Savigny D (2012). Systems thinking for strengthening health systems in LMICs: need for a paradigm shift. Health Policy Plan.

[17] Katahoire AR, Henriksson DK, Ssegujja E, Waiswa P, Ayebare F, Bagenda D (2015). Improving child survival through a district management strengthening and community empowerment intervention: early implementation experiences from Uganda. BMC Public Health.

[18] Ekirapa-Kiracho E, Waiswa P, Rahman M, Makumbi F, Kiwanuka N, Okui O (2011). Increasing access to institutional deliveries using demand and supply side incentives: early results from a quasi-experimental study. BMC Int Health Hum Rights.

[19] Maluka S, Kamuzora P, Sebastian MS, Byskov J, Ndawi B, Hurtig A-K (2010). Improving district level health planning and priority setting in Tanzania through implementing accountability for reasonableness framework : perceptions of stakeholders. BMC Health Serv Res.

[20] Dickovick JT (2012). Decentralization in Uganda: explaining successes and failures in governance (Book-Review). J Polit.

[21] Kyaddondo D, Whyte SR (2003). Working in a decentralized system: a threat to health workers’ respect and survival in Uganda. Int J Health Plann Manage.

[22] Lawn JE, Kinney M, Lee ACC, Chopra M, Donnay F, Paul VK (2009). Reducing intrapartum-related deaths and disability: can the health system deliver?. Int J Gynecol Obstet.

[23] Glasson JB, Chang EM, Bidewell JW (2008). The value of participatory action research in clinical nursing practice. Int J Nurs Pract.

[24] Ekirapa-Kiracho E, Tetui M, Bua J, Kananura RM, Waiswa P, Makumbi F (2017). Maternal and neonatal implementation for equitable systems. A study design paper. Glob Health Action.

[25] Susman G (1983). Action Research: A sociotechnical Systems Perspective.

[26] Conklin J, Kothari A, Stolee P, Chambers L, Forbes D, Le Clair K (2011). Knowledge-to-action processes in SHRTN collaborative communities of practice: a study protocol. Implement Sci.

[27] Uganda Bureau of Statistics (2016). The National Population and Housing Census 2014. Main Report.

[28] Tetui M, Coe A-B, Hurtig A-K, Ekirapa EK, Kiwanuka SN (2017). Stakeholder experiences of using a participatory action research approach to strengthen district local capacity in Eastern Uganda. Glob Health Action.

[29] Ministry of Health (2012). Health Facilities Inventory.

[30] Tetui M, Hurtig A-K, Ekirpa-Kiracho E, Kiwanuka SN, Coe A-B (2016). Building a competent health manager at district level: a grounded theory study from Eastern Uganda. BMC Health Serv Res.

[31] Awortwi N (2011). An unbreakable path? A comparative study of decentralization and local government development trajectories in Ghana and Uganda. Int Rev Adm Sci.

[32] Quinn ER, Faerman SR, Thomson MP, McGrath MR, St Clair LS (2010). Becoming a Master Manager: A Competing Values Approach.

[33] Ovseiko Pavel V, Buchan AM (2012). Organizational culture in an academic health center: an exploratory study using a competing values framework. Acad Med.

[34] Banaszak-Holl J, Castle NG, Lin M, Spreitzer G (2013). An assessment of cultural values and resident-centered culture change in U.S. nursing facilities. Health Care Manage Rev.

[35] Melo RC, Silva MJ, Parreira P (2014). Effective leadership: competing values framework. Procedia Technol.

[36] Patton MQ (2002). Qualitative Research & Evaluation Methods.

[37] Blumer H (1986). Symbolic Interactionism: Perspective and Method.

[38] Dahlgren L, Emmelin M, Winkvist A (2007). Qualitative Methodology for International Public Health.

[39] Braun V, Clarke V (2006). Using thematic analysis in psychology. Qual Res Psychol.

[40] Hartnell CA, Ou AY, Kinicki A (2011). Organizational culture and organizational effectiveness: a meta-analytic investigation of the competing values framework’s theoretical suppositions. J Appl Psychol.

[41] Eerang P, Sangkyun K. Enhancing local community’s involvement and empowerment through practicing Cittaslow: experiences from Goolwa, South Australia. SHS Web Conf. 2014;12:01044.

[42] Thomas P, Mcdonnell J, Mcculloch J, While A, Bosanquet N, Ferlie E (2005). Increasing capacity for innovation in bureaucratic primary care organizations: a whole system participatory action research project. Ann Fam Med.

[43] World Health Organization (2007). Strengthening Management in Low Income Countries: Lessons from Uganda.

[44] Waldau S (2010). Creating Organisational Capacity for Priority Setting in Health Care: Using a Bottom-up Approach to Implement a Top-down Policy Decision.

[45] Pappaioanou M, Malison M, Wilkins K, Otto B, Goodman RA, Churchill RE (2003). Strengthening capacity in developing countries for evidence-based public health: the data for decision-making project. Soc Sci Med.

[46] Wilkins K, Nsubuga P, Mendlein J, Mercer D, Pappaioanou M (2008). The Data for Decision Making project: assessment of surveillance systems in developing countries to improve access to public health information. Public Health.

[47] Struebing J (2010). The SAGE Handbook of Grounded Theory.

