# Supplementary material for: A participatory action research approach to strengthening health managers’ capacity at district level in Eastern Uganda
Source: Health Res Policy Syst. 2017 Dec 28;15(Suppl 2):110. doi: 10.1186/s12961-017-0273-x (PMC5751402; doi:10.1186/s12961-017-0273-x)
Supplement: Additional file 1: — Open peer review reports. (DOCX 21 kb) [file 12961_2017_273_MOESM1_ESM.docx]

**Title**: A participatory action research approach to strengthening health management capacity at district level in Eastern Uganda.

**Reviewer 1:** [Nuggehalli Srinivas Prashanth](https://www.researchgate.net/profile/Nuggehalli_Prashanth)

**Reviewer’s report**

**General comments**

Very few papers exist on participatory action research experiences and hence this is an important contribution to the scarce body of literature. The aim and scope of the paper are well explained. The data analysis approach is also described clearly. My compliments to the authors for undertaking the study and sharing the results. I have 2 minor revisions as described below.

**Minor essential revisions**

There is a need to clarify on how the PAR is or is not a part of the MANIFEST project. How did the MANIFEST project achieve a participatory approach. While this may not be directly the scope of this paper, this is an important context within which the findings of this paper is framed. So, it is important to briefly described the nature of PAR within MANIFEST and how it is being applied. If it is already desecribed elsewhere (in another rpaper for example) the reader could be pointed towards that. The reason for raising this is because the description of the MANIFEST project presented in the first 2 paragraphs do not clearly make linkages to the PAR approach. Also, the paper cited as being one of the outocmes of the intervention package discussion seems to be a quasi-experimental study. So, it is useful to clarify the PAR component of the MANIFEST project.

Positionality of the researcher within this PAR exercise as well as within this study is also important input for the readers to understand this paper.

**Declaration of competing interests**

No conflict of interest to report.

**Reviewer 2:** James W. Begun

**Reviewer’s report**

**General comments**

The case for the importance of PAR at the local level is well-argued. The setting for this research is important and interesting (public sector, Uganda). This is a complex, multi-year, qualitative study with multiple sources of data. The study would be hard to replicate, and positive bias in the interpretation of results is likely. However, several observations resulting from the qualitative methods are useful and interesting, e.g., managers’ desire to work alone to control their work; the need to focus on use of existing resources rather than (potential) external ones.

The paper flows well in terms of organization and logic, and the writing is generally good (some minor editing is required).

The choice of the competing values framework to organize the observations about managerial skills is a good one; the framework has been widely disseminated and utilized in teaching management. The 4 categories of the CVF help to simplify interpretation of complex data. The authors have interesting reflections on the use of participatory approaches at the end of the paper.

**Major compulsory revisions**

p. 3, para 3: I think that the benefits of formal training often are overstated -- consider changing “While the benefits of formal training cannot be overstated,” to “Formal training offers some benefits, but”

p. 6, full para 1: The 7 district level managers and 9 health facility managers are what subset of the total? How were they selected? Are they representative? In this regard, consider moving the “Selection of informants” section into para 1.

I think it would be very difficult to avoid positive bias in the interpretation of these data, as noted on p. 15. Can you expand on the counterinfluence(s)? Or admit the likelihood of positive bias?

**Minor essential revisions**

p.3, para 1: change “is still largely within community interventions” to “is largely restricted to community interventions”

p. 3, para 1: spell out first usage of the acronym PAR

p. 4, full para 3: “combined average population size” is unclear. Is that “combined population size” or just “an average population size”?

p. 5, full para 4: change “complementarily” to “complementary”

p. 5, full para 6: change “workers morale: to “workers’ morale”; change “clients needs” to “clients’ needs.” Several similar changes needed throughout the paper.

p. 6, full para 4: change “committee comprised” to “committee was comprised”; missing period at end of para.

p. 7, full para 2: first sentence is garbled or unclear.

p. 9, para 2: unclear reference to “the two management functions” -- which two?

Table 1 (and Table 3, Table 4): add definition of acronyms, e.g. VHT. Change “Maksph” to “MakSPH”

Table 2: This table would benefit from an improved format. The last row (“Participant observation..”) is difficult to understand. Why is “Interviews with Health managers” bolded?

**Discretionary revisions**

p. 3, para 4: Suggest that you end sentence at “..remedy the situation.” Continue with “The organizations often implement..”

p. 3, para 5: Suggest that you change “formal trainings for health managers further undermine” to “formal training for health managers further undermines”

**Declaration of competing interests**

I declare that I have no competing interests.

**Author’s response to reviews**

| **Respondents’ Guidance** | All revisions are highlighted in the revised version of the paper. |
| --- | --- |
| **Reviewer 1:** [Nuggehalli Srinivas Prashanth](https://www.researchgate.net/profile/Nuggehalli_Prashanth) |  |
| Very few papers exist on participatory action research experiences and hence this is an important contribution to the scarce body of literature. The aim and scope of the paper are well explained. The data analysis approach is also described clearly. My compliments to the authors for undertaking the study and sharing the results. I have 2 minor revisions as described below. | Thank you for the compliments. |
| **Minor essential revisions** |  |
| There is a need to clarify on how the PAR is or is not a part of the MANIFEST project. How did the MANIFEST project achieve a participatory approach.  While this may not be directly the scope of this paper, this is an important context within which the findings of this paper is framed. So, it is important to briefly described the nature of PAR within MANIFEST and how it is being applied. If it is already described elsewhere (in another paper for example) the reader could be pointed towards that. The reason for raising this is because the description of the MANIFEST project presented in the first 2 paragraphs do not clearly make linkages to the PAR approach. Also, the paper cited as being one of the outcomes of the intervention package discussion seems to be a quasi-experimental study. So, it is useful to clarify the PAR component of the MANIFEST project. | We appreciate the comment.  The MANIFEST project was implemented using the PAR approach as a means of increasing chances of strengthening the local health system structures such as management. This was hypothesized to increase chances of sustainability of the project activities and therefore continued improvements in maternal and neonatal health outcomes.  We have made the relationship between PAR and the MANIFEST project more explicit under the section of “brief description of MANIFEST study.” In addition we have provided 2 references for a detailed description of the project design. Page 4. |
| Positionality of the researcher within this PAR exercise as well as within this study is also important input for the readers to understand this paper. | We thank you for pointing this out. The lead research MT, was also the project coordinator of MANIFEST. We acknowledge the bias that could come with this dual role, with the support of the other co-authors, some of whom had no direct involvement with the project, this bias was minimized leading to a more balanced analysis and interpretation of the findings. We have made this explicit in the “methodological consideration” section of the revised paper. Page 15. |
| **Reviewer 2:** James W. Begun |  |
| The case for the importance of PAR at the local level is well argued. The setting for this research is important and interesting (public sector, Uganda). This is a complex, multi-year, qualitative study with multiple sources of data. The study would be hard to replicate, and positive bias in the interpretation of results is likely. However, several observations resulting from the qualitative methods are useful and interesting, e.g., managers’ desire to work alone to control their work; the need to focus on use of existing resources rather than (potential) external ones.  The paper flows well in terms of organization and logic, and the writing is generally good (some minor editing is required).  The choice of the competing values framework to organize the observations about managerial skills is a good one; the framework has been widely disseminated and utilized in teaching management. The 4 categories of the CVF help to simplify interpretation of complex data. The authors have interesting reflections on the use of participatory approaches at the end of the paper. | Thank you for the compliments. |
| **Major compulsory revisions** |  |
| p. 3, para 3: I think that the benefits of formal training often are overstated -- consider changing “While the benefits of formal training cannot be overstated,” to “Formal training offers some benefits, but | We appreciate the comment and the change has been made as suggested. “Formal training offers some benefits but….”  Page 3. |
| p. 6, full para 1: The 7 district level managers and 9 health facility managers are what subset of the total? How were they selected? Are they representative? In this regard, consider moving the “Selection of informants” section into para 1. | The managers were selected purposively and we did not seek to make this representative but the managers were drawn from the three districts in which the MANIFEST intervention was implemented. In addition, while we are not looking for statistical representation, they are representative of the different cadres and districts, which ensured that - maximum variation principles of qualitative research were up held.  We have made this explicit in the methods section and have moved the section of informants selection into paragraph 1 as suggested. Page 6. |
| I think it would be very difficult to avoid positive bias in the interpretation of these data, as noted on p. 15. Can you expand on the counterinfluence(s)? Or admit the likelihood of positive bias? | We acknowledge the concern and have made explicit the possibility of positive bias. Page 15 |
| **Minor essential revisions** |  |
| p.3, para 1: change “is still largely within community interventions” to “is largely restricted to community interventions” | The change has been effected. “….largely restricted to community interventions.. page 3. |
| p. 3, para 1:’ spell out first usage of the acronym PAR | We have written the acronym in full. Participatory Action Research (PAR). Page 3. |
| p. 4, full para 3: “combined average population size” is unclear. Is that “combined population size” or just “an average population size”? | This meant the combined population size average. We have made the correction. Page 4. |
| p. 5, full para 4: change “complementarily” to “complementary” | The change has been effected. “complementary.” Page 5 |
| p. 5, full para 6: change “workers morale: to “workers’ morale”; change “clients needs” to “clients’ needs.” Several similar changes needed throughout the paper. | We appreciate the editorial comments. We have ensured a correction of these and similar mistakes through out the paper. Page 6. |
| p. 6, full para 4: change “committee comprised” to “committee was comprised”; missing period at end of para. | The change has been effected. “...committee was comprised…” page 7 |
| p. 7, full para 2: first sentence is garbled or unclear. | We appreciate the comment. The sentence now reads: An inductive and a deductive approach to thematic analysis was adopted. We hope this is now clear. |
| p. 9, para 2: unclear reference to “the two management functions” -- which two? | We meant the “collaborate” and “control” functions of management. We have made this explicit. Page 9 |
| Table 1 (and Table 3, Table 4): add definition of acronyms, e.g. VHT. Change “Maksph” to “MakSPH” | The acronyms have been written in full.  VHT- village health team  DHT-District health team  DHMT-District health management team.  Maksph-MakSPH  Pages 22, 24, 25. |
| Table 2: This table would benefit from an improved format. The last row (“Participant observation..”) is difficult to understand. Why is “Interviews with Health managers” bolded? | We have improved the explanation on how participant observation was used. Page  Interviews with health managers was bolded by mistake, this has been un bolded. Page 23. |
| **Discretionary revisions** |  |
| p. 3, para 4: Suggest that you end sentence at “..remedy the situation.” Continue with “The organizations often implement..” | The suggestion is appreciated and we have made the changes.  “..agencies to remedy the situation.  “The agencies often implement….”  Page 3. |
| p. 3, para 5: Suggest that you change “formal trainings for health managers further undermine” to “formal training for health managers further undermines” | The suggestion is appreciated and we have effected the change.  “…for health managers further undermines…” page 3. |
